# Safety of a 3-weekly schedule of carboplatin plus pegylated liposomal doxorubicin as first line chemotherapy in patients with ovarian cancer: preliminary results of the MITO-2 randomized trial

**DOI:** 10.1186/1471-2407-6-202

**Published:** 2006-08-01

**Authors:** Sandro Pignata, Giovanni Scambia, Antonella Savarese, Enrico Breda, Paolo Scollo, Rocco De Vivo, Emanuela Rossi, Vittorio Gebbia, Donato Natale, Filomena Del Gaizo, Emanuele Naglieri, Antonella Ferro, Pietro Musso, Alfonso Maria D'Arco, Roberto Sorio, Carmela Pisano, Massimo Di Maio, Giuseppe Signoriello, Annalisa Annunziata, Francesco Perrone

**Affiliations:** 1 Medical Oncology B, National Cancer Institute, Naples, Italy; 2 Gynecologic Oncology Unit, Catholic University of the Sacred Heart, Rome, Italy; 3 Medical Oncology A, Regina Elena Institute, Rome, Italy; 4 Medical Oncology, Fatebenefratelli Hospital, Rome, Italy; 5 Gynecology, Cannizzaro Hospital, Catania, Italy; 6 Medical Oncology, S. Bortolo Hospital, Vicenza, Italy; 7 Medical Oncology C, National Cancer Institute, Naples, Italy; 8 Medical Oncology I, La Maddalena Clinic, Palermo, Italy; 9 Medical Oncology, S. Massimo Hospital, Penne (PE), Italy; 10 Medical Oncology, S. Giuseppe Moscati Hospital, Avellino, Italy; 11 Medical and Experimental Oncology Unit, Oncology Institute, Bari, Italy; 12 Medical Oncology, S. Chiara Hospital, Trento, Italy; 13 Gynecologic Oncology, M. Ascoli Hospital, Palermo, Italy; 14 Medical Oncology, Umberto I Civil Hospital, Nocera Inferiore (SA), Italy; 15 Medical Oncology C, National Cancer Institute – Centro di Riferimento Oncologico, Aviano (PN), Italy; 16 Clinical Trials Unit, National Cancer Institute, Naples, Italy; 17 Department of Medicine and Public health, Second University of Naples, Italy

## Abstract

**Background:**

The MITO-2 (Multicentre Italian Trials in Ovarian cancer) study is a randomized phase III trial comparing carboplatin plus paclitaxel to carboplatin plus pegylated liposomal doxorubicin in first-line chemotherapy of patients with ovarian cancer. Due to the paucity of published phase I data on the 3-weekly experimental schedule used, an early safety analysis was planned.

**Methods:**

Patients with ovarian cancer (stage Ic-IV), aged < 75 years, ECOG performance status ≤ 2, were randomized to carboplatin AUC 5 plus paclitaxel 175 mg/m^2^, every 3 weeks or to carboplatin AUC 5 plus pegylated liposomal doxorubicin 30 mg/m^2^, every 3 weeks. Treatment was planned for 6 cycles. Toxicity was coded according to the NCI-CTC version 2.0.

**Results:**

The pre-planned safety analysis was performed in July 2004. Data from the first 50 patients treated with carboplatin plus pegylated liposomal doxorubicin were evaluated. Median age was 60 years (range 34–75). Forty-three patients (86%) completed 6 cycles. Two thirds of the patients had at least one cycle delayed due to toxicity, but 63% of the cycles were administered on time. In most cases the reason for chemotherapy delay was neutropenia or other hematological toxicity. No delay due to palmar-plantar erythrodysesthesia (PPE) was recorded. No toxic death was recorded. Reported hematological toxicities were: grade (G) 3 anemia 16%, G3/G4 neutropenia 36% and 10% respectively, G3/4 thrombocytopenia 22% and 4% respectively. Non-haematological toxicity was infrequent: pulmonary G1 6%, heart rhythm G1 4%, liver toxicity G1 6%, G2 4% and G3 2%. Complete hair loss was reported in 6% of patients, and G1 neuropathy in 2%. PPE was recorded in 14% of the cases (G1 10%, G2 2%, G3 2%).

**Conclusion:**

This safety analysis shows that the adopted schedule of carboplatin plus pegylated liposomal doxorubicin given every 3 weeks is feasible as first line treatment in ovarian cancer patients, although 37% of the cycles were delayed due to haematological toxicity. Toxicities that are common with standard combination of carboplatin plus paclitaxel (neurotoxicity and hair loss) are infrequent with this experimental schedule, and skin toxicity appears manageable.

## Background

Ovarian cancer has the highest mortality rate of all gynaecologic neoplasms. The high mortality rate may be explained by the lack of symptoms accompanying early disease, resulting in patients being diagnosed at an advanced stage. Furthermore, long term results obtained with current treatments are limited.

The combination of paclitaxel and cisplatin became standard first line chemotherapy following the results of the GOG-111 [1] and subsequent confirmatory trials [2], because it was more effective than the combination of cyclophosphamide and cisplatin. Due to its more favourable toxicity profile, paclitaxel combined with carboplatin has replaced paclitaxel and cisplatin as the standard first line chemotherapy worldwide [3-5]. Debulking surgery and first line systemic chemotherapy induce complete or partial response in up to 80% of patients, with about a 25% pathological complete remission rate [4-6]. Unfortunately, recurrences occur in the majority of patients, and the 5-year survival rate is only 30–50%, largely depending on the initial FIGO stage.

Anthracyclines were originally used in the first line treatment of ovarian cancer in the '70s, when *in vitro *experiments showed a dose-response relationship in ovarian cancer cell lines, and activity against epithelial ovarian cancer was subsequently proven in clinical trials [7-9]. The role of anthracyclines in ovarian cancer, though still debated, has had renewed interest after the availability of liposomal antracyclines.

Pegylated liposomal doxorubicin (PLD) is a formulation of doxorubicin encapsulated in liposomes in order to obtain pharmacokinetic properties not available with conventional formulation of the drug: lower plasma concentration peak, lower clearance, smaller distribution volume, longer half-life and higher AUC, resulting in a different toxicity profile [10-15]. The size of the liposomes allows selective accumulation in the tumor vascular bed following extravasation through the leaky tumor vasculature [11,12]. In addition, the special coating (pegylation) of the liposomes is associated with reduced clearance by the mononuclear phagocyte system, thus helping to maintain active drug concentrations for longer periods [10-13].

A phase III randomised trial [16] compared PLD with standard topotecan, in second line treatment of ovarian cancer. A 5-year update of this trial has been recently published [17], and PLD proved to be statistically superior to topotecan in terms of overall survival. Furthermore, the analysis conducted in the subgroup of "platinum-sensitive" patients showed a particularly significant advantage for PLD compared to topotecan in this group. Following these results, PLD is now considered the drug of choice for the treatment of relapsed ovarian cancer in terms of activity, toxicity and cost benefits. A phase II trial has recently shown that the combination of carboplatin and PLD given every 4 weeks is highly effective in recurrent platinum-sensitive ovarian cancer [18]. These data represent a strong rationale for testing PLD in the first line treatment of ovarian cancer.

The MITO-2 (Multicentre Italian Trials in Ovarian cancer) study is a randomized phase III study comparing carboplatin plus paclitaxel to carboplatin plus PLD in first-line treatment of ovarian cancer patients. The primary end-point is progression-free survival. The secondary end-points are toxicity, objective response rate, quality of life and overall survival. In both arms chemotherapy is administered every 3 weeks. Due to the paucity of published safety data on the 3-weekly schedule adopted for the combination of carboplatin and PLD, an early safety analysis had been planned and the results are reported in this paper.

## Methods

Patient randomization in the MITO-2 trial started in January 2003. The protocol was approved by the Ethics Committee of each participating center. Written informed consent was obtained from each enrolled patient, prior to study entry. Patients with cytologic or histological diagnosis of epithelial ovarian carcinoma (stage Ic-IV), and an Eastern Cooperative Oncology Group (ECOG) performance status ≤ 2 were eligible. Exclusion criteria were age ≥ 75 years, prior or concurrent malignant cancer (except for non-melanoma skin cancer and for *in situ *carcinoma of the uterine cervix, if adequately treated), brain metastases, inadequate bone marrow function (neutrophils <2,000/mm^3 ^or platelets <100,000/mm^3^); abnormal renal function (total serum creatinine level > 1.25 the upper normal limit), abnormal liver function (sAST or sALT or total serum bilirubin levels > 1.25 the upper normal limit, except if caused by liver metastases), heart disease (heart failure, heart attack in the previous 6 months, atrio-ventricular block of any degree, serious arrhythmia).

Patients in the standard arm received carboplatin, area under curve (AUC) 5, intravenously (i.v.), plus paclitaxel, 175 mg/m^2^, i.v. in a 3-hour infusion, both drugs on day 1, every 21 days. Patients in the experimental arm received carboplatin AUC 5 i.v. and PLD [Caelyx^®^], 30 mg/m^2^, both drugs on day 1, every 21 days. Chemotherapy was administered for a maximum of 6 cycles. Carboplatin was dosed in accordance with the Calvert formula [19], and administered in 250 ml physiologic solution, over 30 minutes. PLD was administered after carboplatin infusion, in 250 ml 5% glucosate solution, over 1 hour.

Treatment toxicity and adverse events were coded according to the Common Toxicity Criteria (CTC) of the National Cancer Institute (NCI), version 2.0 [20].

Criteria for retreatment were: neutrophils ≥ 1,500/mm^3^, platelets ≥ 100,000/mm^3^, Hgb ≥ 9 g/dl, absence of organ toxicity ≥ 2 (with the exclusion of hair loss). If these minimum conditions were not met, the cycle was postponed by 7 days for a maximum of 2 weeks. If the treatment was delayed for more than 2 weeks, chemotherapy was discontinued due to unacceptable prolonged toxicity. After the first 3 cycles, in the absence of unacceptable toxicity, patients with objective response or stable disease received a further 3 cycles, for a maximum number of six cycles.

A 20% dose reduction was planned for grade 4 neutropenia lasting more than 7 days, grade 3 thrombocytopenia lasting more than 7 days, or neurotoxicity. In case of creatinine clearance < 60 ml, the dose of carboplatin was reduced from AUC 5 to AUC 4. In case of grade 2 or higher palmar-plantar erythrodysesthesia (PPE), chemotherapy was delayed for up to 2 weeks, until recovery to grade 0–1, and then resumed with a 25% dose reduction. If cutaneous toxicity had not recovered after 2 weeks, PLD was withdrawn.

No prophylactic use of G-CSF was recommended. Therapeutic and prophylactic use of G-CSF was allowed for febrile or afebrile grade 4 neutropenia.

Complete blood counts were performed at baseline and weekly. Laboratory exams (sAST, sALT, total serum protein, albumin, bilirubinaemia, alkaline phosphatase, lactate dehydrogenase, creatininaemia, blood urea nitrogen, glycaemia, uricaemia, serum electrolytes) and urinalysis were planned baseline, and then repeated before each cycle and 3 weeks after the end of the last cycle.

A preplanned safety analysis was performed in July 2004. The safety analysis was planned to be descriptive and was not driven by a pre-stated hypothesis and consequent statistical plan. A sample size of 50 patients was arbitrarily chosen, and the first 50 patients assigned to CLD arm and receiving at least one dose of experimental drugs were considered for this safety analysis.

## Results

Main baseline characteristics of the patients are reported in table 1. Median age was 60 years (range 34–75). All but two patients had a good ECOG performance status (0 or 1). Almost half of the patients were optimally debulked.

Forty-three patients (86%) completed the planned number of cycles. Of the remaining seven patients, chemotherapy was interrupted before completion for progressive disease or worsening of disease symptoms (5 cases), for patient's refusal (1) and for prolonged toxicity (1).

Three patients interrupted pegylated liposomal doxorubicin, continuing carboplatin, after the first dose: 1 patient for grade 3 allergy, and 2 patients for prolonged neutropenia. Four patients had a dose reduction of liposomal doxorubicin because of myelotoxicity (3 patients after 4 cycles, and 1 patient after 5 cycles).

Thirty-four patients (68%) delayed at least one cycle due to toxicity. Reasons for chemotherapy delays are detailed in table 2. In most cases, the reason was sustained neutropenia or other hematological toxicity. No delay due to cutaneous toxicity was recorded. Overall, 37% of the cycles were delayed.

Details of worst haematological and non-haematological toxicities are reported in table 3. No toxic death was recorded. Grade 3 anemia was reported in 8 patients (16%), with 6 patients receiving transfusions. Grade 3/4 neutropenia was observed in 36% and 10% of patients respectively. Grade 3/4 thrombocytopenia occurred in 22% and 6% of patients respectively, and in all cases was asymptomatic and did not require platelet transfusion. Allergy was reported in 5 patients (10%), leading to treatment withdrawal in 1 case.

Organ toxicity was infrequent: grade 1 pulmonary in 3 patients (6%), grade 1 heart rhythm in 2 patients (4%), liver toxicity in 6 patients (grade 1, 6%; grade 2, 4%; and grade 3, 2%). Complete hair loss was reported in 3 patients. Only 1 patient experienced grade 1 neuropathy. Palmar-plantar erythrodysesthesia was recorded in 14% of the patients (grade 1, 10%; grade 2, 2%; grade 3, 2%).

The phase III trial is ongoing; as of January 2006, 395 patients have been enrolled.

## Discussion

Pegylated liposomal doxorubicin is considered to be one of the more active drugs in ovarian cancer. This formulation has significant advantages in terms of tolerability compared to conventional doxorubicin: the most frequent toxicities consist of cutaneous and mucosal toxicity (hand-foot syndrome and stomatitis), with a very low rate of nausea, hair loss, extravasation-related necrosis or reduction in the ventricular ejection fraction [15-17]. When given as a single agent, the drug is usually administered every 4 weeks. A phase II trial conducted in France, the results of which were first presented at ASCO 2004 [18], analysed the combination of PLD and carboplatin, in patients with platinum-sensitive recurrent ovarian cancer. Carboplatin was administered at AUC 5, and liposomal doxorubicin at the dose of 30 mg/m^2^, both drugs given every 4 weeks. The combination showed a very high rate of activity (objective response rate 68%), and a favourable toxicity profile.

The proven efficacy of liposomal doxorubicin in second line treatment, and the possibility of easily combining it with carboplatin, encouraged us to test the efficacy of the combination of carboplatin and pegylated liposomal doxorubicin in first line treatment of patients with ovarian tumour in a phase III multicentric trial comparing it with standard chemotherapy (carboplatin plus paclitaxel). In order to maintain the full dose of carboplatin we chose a 3-weekly schedule of carboplatin and PLD.

Our data show that this schedule is feasible and has a favourable toxicity profile. Anemia, thrombocytopenia, and neutropenia were the most frequent toxicities, but no case of febrile neutropenia was recorded, and no case of thrombocytopenia was symptomatic. Though 68% of the patients had at least one cycle delayed, most often due to persistent neutropenia at day 21, most of the cycles were still given on time. Non-hematological toxicity of the combination appears to be different from the toxicity expected with carboplatin plus paclitaxel, with markedly less neurotoxicity and hair loss, and a higher incidence of mild skin toxicity. The limited incidence and severity of PPE clearly indicates that skin toxicity is significantly less frequent at this dose compared to single agent PLD when given at higher doses. Overall, our toxicity data compare favourably with the results reported in the literature in platinum-sensitive recurrent disease [18,21]. The main toxic effect recorded by the French group was myelosuppression, with the same proportion of grade 3–4 neutropenia, and slightly more anemia (grade 3 34%), and thrombocytopenia (grade 3–4 31%), probably as a consequence of the second-line setting. delay in the administration of chemotherapy was necessary in that study, although less frequently (30% patients) due to the every 4-weeks schedule.

Our study shows that the non-hematological toxicity profile of carboplatin and PLD is particularly safe. The hematologic toxicities are associated with a significant number of delays in chemotherapy administration for neutropenia and thrombocytopenia, though no cases of febrile neutropenia or symptomatic thrombocytopenia were reported. The low rate of neurotoxicity is interesting, given that neurotoxicity is a major factor in non compliance with standard first line chemotherapy. In addition, the low rate of hair loss could represent a significant advantage compared to the standard regimen of carboplatin plus paclitaxel.

## Conclusion

The combination of carboplatin and pegylated liposomal doxorubicin, in an every-3-week schedule, can be safely given as first line treatment of patients with ovarian cancer. The MITO-2 study continues the planned enrollment, and its results will provide useful information regarding the future role of this combination for these patients.

## Competing interests

Sandro Pignata and Francesco Perrone received in the past five years grants from Schering Plough. The other Authors declare that they have no competing interests.

## Authors' contributions

SP and FP projected the trial, participated in its design and coordination;

SP, MDM and FP drafted the manuscript;

SP, GS, AS, EB, PS, RDV, ER, VG, DN, FDG, EN, EG, PM, AMDA, RS and CP treated the patients, collected the clinical data useful for the analysis and revised the article critically for important intellectual content;

MDM, FP, GS and AA performed the analysis of the data.

All authors read and approved the final manuscript.

## Appendix

Participating MITO-2 Institutions and coauthors

National Cancer Institute, Naples (Medical Oncology B: S Pignata, C Pisano, RV Iaffaioli; Ginecology: S Greggi; Medical Oncology C: E Rossi; Clinical Trials Unit: F Perrone, M Di Maio, E De Maio, A Morabito, G De Feo, R D'Aniello);

Department of Medicine and Public health, Second University of Naples, Naples, Italy (C Gallo, G Signoriello, A Annunziata, P Chiodini);

Gynecologic Oncology Unit, Catholic University of the Sacred Heart, Rome-Campobasso (G Scambia, G Ferrandina, D Lorusso, A Di Stefano);

Medical Oncology A, Regina Elena Institute, Rome, Italy (A Savarese, F Cognetti, A. Felici);

Medical Oncology C, National Cancer Institute – Centro di Riferimento Oncologico, Aviano (PN), Italy (R Sorio, S Scalone, E Campagnutta);

Medical Oncology, Fatebenefratelli Hospital, Rome, Italy (E Breda, V Zagonel, R Bompiani);

Malzoni Clinic, Avellino (C Malzoni, M Malzoni, A. Vernaglia Lombardi);

Medical Oncology I, La Maddalena Clinic, Palermo, Italy (V Gebbia, R Agueli, A Testa);

Gynecologic Oncology, M. Ascoli Hospital, Palermo, Italy (P Musso, L Segreto, R Demma);

Gynecology, Cannizzaro Hospital, Catania, Italy (P Scollo, G Scibilia, E Lomeo);

Medical Oncology, Ospedali Riuniti, Reggio Calabria, Italy (M Nardi, P Del Medico, D Azzarello);

Medical Oncology, S. Chiara Hospital, Trento, Italy (E Galligioni, A Ferro);

Oncology Institute, Bari, Italy (V Lorusso, A Latorre);

Medical Oncology, S. Bortolo Hospital, Vicenza, Italy (R De Vivo);

Medical Oncology, Umberto I Civil Hospital, Nocera Inferiore (SA), Italy (AM D'Arco, A Fabbrocini);

Bellaria Hospital, Bologna, Italy (L Crinò, S Rimondini);

Ramazzini Hospital, Carpi (MO), Italy (F Artioli, L Scaltriti);

Medical Oncology, Fatebenefratelli Hospital, Benevento, Italy (A Febbraro, MC Merola);

Medical Oncology, S. Massimo Hospital, Penne (PE), Italy (D Natale, C Chiapperino);

Civil Hospital, Faenza (RA), Italy (S Tamberi, A Gambi);

Gynecologic Unit CHC, Maternidade Bissaya – Barreto, Coimbra, Portugal (MO Campos, IM Henriques);

University of Palermo, Policlinico Giaccone, Palermo, Italy (N Gebbia, MR Valerio);

Medical Oncology, Carlo Poma Hospital, Mantova, Italy (E Aitini, G Cavazzini);

Ospedale degli Infermi, Rimini – Cattolica, Italy (A Ravaioli, G Oliverio);

Gynecology, Ospedali Riuniti, Bergamo, Italy (L Frigerio, L Carlini);

Medical Oncology, University of Cagliari, Cagliari, Italy (B Massidda);

Ginecology, S Anna Hospital, Turin, Italy (S Danese);

Medical Oncology, S. Giuseppe Moscati Hospital, Avellino, Italy (F Del Gaizo);

Medical and Experimental Oncology Unit, Oncology Institute, Bari, Italy (E Naglieri);

Medical Oncology, Mariano Santo Hospital, Cosenza, Italy (R Biamonte);

Miulli hospital, Acquaviva delle Fonti (BA), Italy (G Nettis);

S. Anna Hospital, Ferrara, Italy (M Marzola);

Cotugno Hospital, Naples, Italy (V Montesarchio);

Budrio Bentivoglio, Italy (V Arigliano);

Pierantoni Hospital, Forlì, Italy (N Riva);

Gynecology, Second University of Naples, Italy (GC Balbi).

## Pre-publication history

The pre-publication history for this paper can be accessed here:



## Figures and Tables

**Table 1:** Baseline characteristics of patients (n = 50)

**Age (years)**	Median	60	
	Range	34–76	
**Residual disease**	Absent	17	(34%)
	≤ 1 cm	6	(12%)
	> 1 cm	23	(46%)
	Surgery not performed	4	(8%)

**Stage**	IC	3	(6%)
	II	6	(12%)
	III	29	(58%)
	IV	12	(24%)

**PS**	0	30	(60%)
	1	18	(36%)
	2	2	(4%)

**Grading**	1	2	(4%)
	2	14	(28%)
	3	22	(44%)
	4	12	(24%)

**Histologic type**	Serous	30	(60%)
	Mucinous	3	(6%)
	Endometrioid	7	(14%
	Undifferentiated	3	(6%)
	Clear cell	1	(2%)
	Mixed	4	(8%)
	Other	2	(4%)

PS = Performance Status

**Table 2:** Causes of chemotherapy delays due to toxicity

	**Cycle 2 (49 pts)**	**Cycle 3 (47 pts)**	**Cycle 4 (46 pts)**	**Cycle 5 (44 pts)**	**Cycle 6 (43 pts)**	**Overall**
Neutropenia	10	11	3	7	8	**39**
Thrombocytopenia	-	4	2	4	4	**14**
Neutropenia + Thrombocytopenia	2	2	5	3	3	**15**
Anemia	-	2	1	-	1	**4**
Anemia + Neutropenia + Thrombocytopenia	-	-	1	-	1	**2**
Liver toxicity	1	1	2	1	1	**6**
Fever	-	-	-	1	-	**1**
Other (not specified)	1	-	-	1	4	**6**
Total	14 (28.6%)	20 (42.6%)	14 (30.4%)	17 (38.6%)	22 (51.2%)	**87/229 (37%)**

**Table 3:** Worst toxicity per patient (n = 50)

**Toxicity**	**Worst NCI – CTC grade **Number of patients (%)					
	
	**0**	**1**	**2**	**3**	**4**	**5**
Anemia	15 (30%)	8 (16%)	19 (38%)	8 (16%)	-	-
Leukopenia	12 (24%)	11 (22%)	19 (38%)	6 (12%)	2 (4%)	-
Neutropenia	11 (22%)	5 (10%)	11 (22%)	18 (36%)	5 (10%)	-
Febrile neutropenia	50 (100%)	-	-	-	-	-
Neutropenic infection	50 (100%)	-	-	-	-	-
Non neutrop. infection	49 (98%)	-	1 (2%)	-	-	-
Platelets	25 (50%)	7 (14%)	5 (10%)	11 (22%)	2 (4%)	-
Platelet transfusion	50 (100%)	-	-	-	-	-
RBC transfusion	47 (94%)	-	-	3 (6%)	-	-
Allergy	45 (90%)	2 (4%)	1 (2%)	2 (4%)	-	-
Bleeding	50 (100%)	-	-	-	-	-
Fatigue	27 (54%)	13 (26%)	10 (20%)	-	-	-
Heart rhythm	48 (96%)	2 (4%)	-	-	-	-
Cardiovascular	48 (96%)	1 (2%)	-	1 (2%)	-	-
Pulmonary	47 (94%)	3 (6%)	-	-	-	-
Fever	48 (96%)	2 (4%)	-	-	-	-
Weight loss	48 (96%)	1 (2%)	1 (2%)	-	-	-
Hair loss	40 (80%)	7 (14%)	3 (6%)	-	-	-
Local reaction	49 (98%)	1 (2%)	-	-	-	-
Skin (including PPE)	43 (86%)	5 (10%)	1 (2%)	1 (2%)	-	-
Anorexia	44 (88%)	4 (8%)	2 (4%)	-	-	-
Constipation	35 (70%)	9 (18%)	6 (12%)	-	-	-
Diarrhoea	49 (98%)	1 (25)	-	-	-	-
Nausea	27 (54%)	16 (32%)	6 (12%)	1 (2%)	-	-
Vomiting	38 (76%)	8 (16%)	2 (4%)	2 (4%)	-	-
Stomatitis	42 (84%)	4 (8%)	4 (8%)	-	-	-
Liver	44 (88%)	3 (6%)	2 (4%)	1 (2%)	-	-
Neuropathy	49 (98%)	1 (2%)	-	-	-	-
Kidney	49 (98%)	1 (2%)	-	-	-	-
Other	47 (94%)	2 (4%)*	-	1 (2%)**	-	-

RBC = red blood cell; PPE = palmar-plantar erythrodysesthesia

*hyperglycemia (1 patient); epigastric pain (1 patient); ** abdominal pain

## References

[B1] Mc Guire WP, Hoskins WJ, Brady MF, Kucera PR, Partridge EE, Look KY, Clarke-Pearson DL, Davidson M (1996). Cyclophosphamide and cisplatin compared with placlitaxel and cisplatin in patients with stage III and stage IV ovarian cancer. N Engl J Med.

[B2] Piccart MJ, Bertelsen K, James K, Cassidy J, Mangioni C, Simonsen E, Stuart G, Kaye S, Vergote I, Blom R, Grimshaw R, Atkinson RJ, Swenerton KD, Trope C, Nardi M, Kaern J, Tumolo S, Timmers P, Roy JA, Lhoas F, Lindvall B, Bacon M, Birt A, Andersen JE, Zee B, Paul J, Baron B, Pecorelli S (2000). Randomized intergroup trial of cisplatin-paclitaxel versus cisplatin-cyclophosphamide in women with advanced epithelial ovarian cancer: three-year results. J Natl Cancer Inst.

[B3] Ozols RF, Bundy BN, Greer BE, Fowler JM, Clarke-Pearson D, Burger RA, Mannel RS, DeGeest K, Hartenbach EM, Baergen R, Gynecologic Oncology Group (2003). Phase III trial of carboplatin and paclitaxel compared with cisplatin and paclitaxel in patients with optimally resected stage III ovarian cancer: a Gynecologic Oncology Group study. J Clin Oncol.

[B4] Neijt JP, Engelholm SA, Tuxen MK, Sorensen PG, Hansen M, Sessa C, de Swart CA, Hirsch FR, Lund B, van Houwelingen HC (2000). Exploratory phase III study of cisplatin and paclitaxel versus carboplatin and paclitaxel in advanced ovarian cancer. J Clin Oncol.

[B5] du Bois A, Luck HJ, Meier W, Adams HP, Mobus V, Costa S, Bauknecht T, Richter B, Warm M, Schroder W, Olbricht S, Nitz U, Jackisch C, Emons G, Wagner U, Kuhn W, Pfisterer J, Arbeitsgemeinschaft Gynakologische Onkologie Ovarian Cancer Study Group (2003). A randomized clinical trial of cisplatin/paclitaxel versus carboplatin/paclitaxel as first-line treatment of ovarian cancer. J Natl Cancer Inst.

[B6] Bristow RE, Tomacruz RS, Armstrong DK, Trimble EL, Montz FJ (2002). Survival effect of maximal cytoreductive surgery for advance ovarian cancer in the platinum era: a metanalysis. J Clin Oncol.

[B7] Ozols RF, Willson JK, Weltz MD, Grotzinger KR, Myers CE, Young RC (1980). Inhibition of human ovarian cancer colony formation by Adriamicin and its metabolites. Cancer Res.

[B8] Ovarian Cancer Meta-Analysis Project (1991). Cyclophosphamide plus cisplatin versus cyclophosphamide, doxorubicin and cisplatin chemotherapy of ovarian carcinoma. J Clin Oncol.

[B9] A'Hern R, Gore ME (1995). The impact of doxorubicin on survival in advanced ovarian cancer. J Clin Oncol.

[B10] Vaage J, Donovan D, Mayhew E, Abra R, Huang A (1993). Therapy of human ovarian carcinoma xenografts using doxorubicin encapsulated in sterically stabilized liposomes. Cancer.

[B11] Sakakibara T, Chen FA, Kida H, Kunieda K, Cuenca RE, Martin FJ, Bankert RB (1996). Doxorubicin encapsulated in strerically stabilized liposomes is superior to free drug or drug-containing conventional liposomes at suppressing growth and metastases of human lung tumors xenografts. Cancer Res.

[B12] Vaage J, Donovan D, Uster P, Working P (1997). Tumor uptake of doxorubicin in polyethylene glycol-coated liposomes and therapeutic effect against a xenografted human pancreatic carcinoma. Br J Cancer.

[B13] Muggia FM, Hainsworth JD, Jeffers S, Miller P, Groshen S, Tan M, Roman L, Uziely B, Muderspach L, Garcia A, Burnett A, Greco FA, Morrow CP, Paradiso LJ, Liang LJ (1997). Phase II study of liposomal doxoribicin in refractory ovarian cancer. Antitumor activity and toxicity modification by liposomal encapsulation. J Clin Oncol.

[B14] Gordon AN, Granai CO, Rose PG, Hainsworth J, Lopez A, Weissman C, Rosales R, Sharpington T (2000). Phase II study of liposomal doxorubicin in platinum-paclitaxel refractory epithelial ovarian cancer. J Clin Oncol.

[B15] Safra T, Groshen S, Jeffers S, Tsao-Wei DD, Zhou L, Muderspach L, Roman L, Morrow CP, Burnett A, Muggia FM (2001). Treatment of patients with ovarian carcinoma with pegylated liposomal doxorubicin. Analysis of toxicities and predictors of outcome. Cancer.

[B16] Gordon AN, Fleagle JT, Guthrie D, Parkin DE, Gore ME, Lacave AJ (2001). Recurrent epithelial ovarian carcinoma: a randomized phase III study of pegylated liposomal doxorubicin versus topotecan. J Clin Oncol.

[B17] Gordon AN, Tonda M, Sun S, Rackoff W, Doxil Study 30–49 Investigators (2004). Long-term survival advantage for women treated with pegylated liposomal doxorubicin compared with topotecan in a phase 3 randomized study of recurrent and refractory epithelial ovarian cancer. Gynecol Oncol.

[B18] Ferrero JM, Weber B, Lepille D, Orfeuvre H, Combe M, Mayer F, Leduc B, Bourgeois H, Paraiso D, Pujade-Lauraine E (2004). Caelyx and Carboplatin in patients with advanced ovarian cancer in late relapse (>6 months): late results of a GINECO phase II trial [abstract]. Proc Am Soc Clin Oncol.

[B19] Calvert AH, Newell DR, Gumbrell LA, O'Reilly S, Burnell M, Boxall FE, Siddik ZH, Judson IR, Gore ME, Wiltshaw E (1989). Carboplatin dosage: prospect of evaluation of a simple formula based on renal function. J Clin Oncol.

[B20] National Cancer Institute – Cancer Therapy Evaluation Program: Common Toxicity Criteria. Version 2.0 April 30, 1999. http://ctep.info.nih.gov.

[B21] du Bois A, Burges A, Meier W, Pfisterer J, Schmalfeldt B, Richter B, Jackisch C, Staehle A, Kimmig R, Elser G (2006). Pegylated liposomal doxorubicin and carboplatin in advanced gynecologic tumors: a prospective phase I/II study of the Arbeitsgemeinschaft Gynaekologische Onkologie Studiengruppe Ovarialkarzinom (AGO-OVAR). Ann Oncol.

